# Clinical and Laboratory Features of Hemophagocytic Lymphohistiocytosis in People With Disseminated Histoplasmosis

**DOI:** 10.1093/ofid/ofaf602

**Published:** 2025-09-30

**Authors:** Burton Mandrell, Tatsiana Savenka, Michael Saccente

**Affiliations:** Division of Infectious Diseases and Geographic Medicine, University of Texas Southwestern Medical Center, Dallas, Texas, USA; Department of Internal Medicine, Division of Infectious Diseases, University of California-Davis Health, Sacramento, California, USA; Department of Internal Medicine, Division of Infectious Diseases, University of Arkansas for Medical Sciences and the Central Arkansas Veterans Healthcare System, Little Rock, Arkansas, USA

**Keywords:** 1;3-beta-Dglucan, disseminated histoplasmosis, hemophagocytic lymphohistiocytosis, hyperferritinemia

## Abstract

**Background:**

Hemophagocytic lymphohistiocytosis (HLH) is a life-threatening syndrome involving pathologic excitation of the immune system. Disseminated histoplasmosis (DH) is a known trigger of HLH. However, the prevalence of HLH in DH is unknown. Limited data exist on risk factors and outcomes. The goals of this study are to determine the prevalence of HLH among participants with DH at a single center, identify risk factors for HLH in this population, and describe the treatment and outcomes of people with DH and HLH.

**Methods:**

We retrospectively identified cases of DH at our institution from 2014 to 2022 and reviewed electronic medical records. We used HLH-2004 criteria to identify those with HLH.

**Results:**

Among 110 participants with DH, 22 (20%) met criteria for HLH. In the subset who were hospitalized, 24% (22/93) had HLH. Compared to participants without HLH, the HLH cohort was more likely to have serum ferritin above the limit of quantification (LOQ) (>15 000 ng/ml), urine *Histoplasma* antigen above the LOQ (>19 ng/mL), serum 1,3 beta-D-glucan (BDG) above the LOQ (>500 pg/mL), and more likely to require intensive care. There was no significant difference in HIV/AIDS status, race, or sex. Mortality was numerically higher in the HLH cohort (18% vs 7%), but the difference was not statistically significant.

**Conclusions:**

Nearly a quarter of participants with DH admitted to our hospital had HLH. Extreme levels of serum ferritin, urine *Histoplasma* antigen, and serum BDG should prompt investigation for HLH. Further studies are needed to assess optimal treatment strategies.

## Teaser

Hemophagocytic lymphohistiocytosis occurred in 20% of people with disseminated histoplasmosis at our institution in Arkansas. Very elevated serum ferritin, beta-D-glucan, and urine *Histoplasma* antigen correlated with HLH and should prompt further investigation for HLH.

Hemophagocytic lymphohistiocytosis (HLH) is a life-threatening, hyperinflammatory syndrome of pathologic over-activation of T cells and macrophages. Historically, HLH is subdivided into primary and secondary forms. Primary HLH is attributed to inherited genetic mutations seen in pediatric patients; whereas secondary HLH is typically described in adults with an identifiable trigger, usually infection, malignancy, or autoimmune disease [1].

Among adults with HLH, 50% are associated with infection [2]. Implicated pathogens include herpesviruses, HIV-1, and a variety of intracellular bacteria, protozoa, and fungi, including mycobacteria, *Leishmania, Babesia,* and *Histoplasma* [2]. Infection-associated HLH has typically been described in adults who are immunocompromised or immunosuppressed: especially people with HIV/AIDS, solid organ transplants, hematopoietic stem-cell transplants, or those receiving immune modulating therapies [3, 4]. A review of HLH in people with HIV included 81 cases of HLH, 17% of which were associated with histoplasmosis [4]. In a study from Mexico, 36% of 72 people with HIV and disseminated histoplasmosis (DH) met diagnostic criteria for HLH [5]. However, the prevalence of HLH associated with DH in the United States remains incompletely characterized, and risk factors for HLH in the setting of histoplasmosis are poorly defined.

The distribution of *Histoplasma* infections in the United States has expanded beyond its historical area of endemicity, and the number of *Histoplasma* infections is increasing with approximately 80 000 *Histoplasma* infections from 2007 to 2016 per Medicare claims review [6]. The number of hospitalizations associated with histoplasmosis nearly doubled from 2001 to 2012 [7]. As the number of *Histoplasma* infections increases, awareness and recognition of histoplasmosis and its complications, such as HLH, will become more important. Arkansas is located within the traditional histoplasmosis-endemic region. Every county in Arkansas has an incidence of greater than 100 cases/100 000 person-years, and some have rates greater than 1000 cases/100 000 person-years [6].

An optimal treatment regimen for infection-associated HLH is not known. The original HLH-1994 protocol included treatment with 6 weeks of etoposide and dexamethasone [8]. Consensus statements for treatment of HLH in adults from the Histiocyte Society in 2019 support directed antimicrobial treatment for intracellular pathogens, reserving cytotoxic therapies for relapsed or refractory HLH [9]. However, organism specific studies have shown a discordance in treatment regimens. HLH due to visceral leishmaniasis in children was treated with amphotericin B alone [10, 11]. On the other hand, EBV-associated HLH usually requires cytotoxic treatment with etoposide, dexamethasone, rituximab, and even hematopoietic stem-cell transplant [12, 13]. The use of HLH directed therapies in people with DH is poorly characterized. A 2025 systematic review of HLH in invasive fungal infections (including histoplasmosis) in people without HIV observed no statistically significant difference in 30-day mortality in people who received HLH directed therapy versus people who did not; however, the medications, dosing, and duration of HLH therapies were not defined [3]. Novel cytokine targeted therapies for HLH, including anakinra, emapalumab, ruxolitinib, and tocilizumab, are active areas of research [14–16]. The balance of physiologic versus pathologic immune response in the setting of infection adds a layer of complexity to clinical decisions regarding immune modulating interventions. Treatment decisions are typically made on a case-by-case basis, but more research is needed.

## METHODS

We used the Arkansas Clinical Data Repository at the University of Arkansas for Medical Sciences for retrospective identification of cases of histoplasmosis, based on ICD code B39, seen at our institution between 1 January 2014 and 31 December 2022. We reviewed each medical record to identify participants with DH. We defined DH as: (1) culture of *Histoplasma capsulatum* from a nonpulmonary site, or (2) nonpulmonary tissue histopathology with yeast identifiable as *Histoplasma*, or (3) A combination of clinical abnormalities consistent with DH *plus* a positive *Histoplasma* antigen enzyme immunoassay in urine (MiraVista Diagnostics, Indianapolis, IN). Typical clinical findings in each case included most or all of the following: fever, night sweats, fatigue, unintentional weight loss, hepatosplenomegaly, lymphadenopathy, mucosal ulcerations, skin lesions, pancytopenia, and elevated alkaline phosphatase.

Participants were included if they met diagnostic criteria of DH and were over the age of 18 at time of diagnosis. HLH was diagnosed by meeting 5 out of 8 HLH-2004 criteria: fever, splenomegaly, bicytopenia or pancytopenia (hemoglobin < 9 g/dL, absolute neutrophil count < 1000/µL, and platelet count < 100 000/µL), hypertriglyceridemia >265 mg/dL or hypofibrinogenemia < 150 mg/dL, hyperferritinemia > 500 ng/mL, hemophagocytosis noted on tissue biopsy (bone marrow, spleen, or lymph node), low or absent NK cell activity, and elevated soluble IL-2 receptor (also known as soluble CD25). Splenomegaly was assumed measured and negative if not explicitly positive on imaging or physical exam. Participants were only diagnosed with HLH by meeting at least 5 HLH-2004 criteria. Participants with less than 5 criteria evaluated were classified as non-HLH.

The Fungitell Assay (Associates of Cape Cod, East Falmouth, MA) determined 1,3 beta-D-glucan (BDG) levels as follows: negative, <60 pg/mL; indeterminate, 60–79 pg/mL, and positive, ≥80 pg/mL. Values <31 pg/mL and those >500 pg/mL were censored at 30 pg/mL and 500 pg/mL, respectively. The upper limit of quantification of serum ferritin is 15 000 ng/mL at our institution, and values were censored at 15 000 if reported as >15 000. *Histoplasma* antigens above 19 pg/mL, the upper limit of quantification, were censored at 19.

Continuous variables are presented as medians with interquartile ranges. Categorical variables, shown as counts and percentages, are compared with Fisher's exact test or Mann–Whitney U test. We used odds ratios to identify potential predictors of HLH. All tests of significance were 2-tailed, and *P* values <.05 were considered statistically significant.

## RESULTS

**Figure 1.**
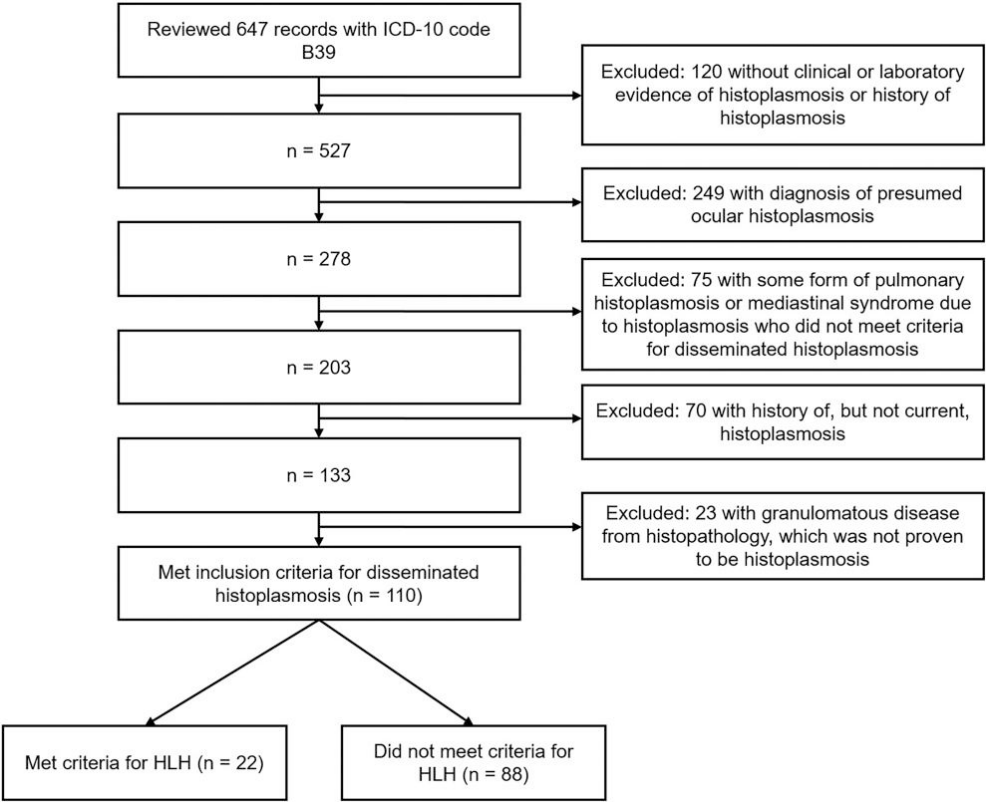
Selection and exclusion criteria for participants with disseminated histoplasmosis.

In total, we reviewed 647 records to identify 110 people who met criteria for DH (Figure 1). The majority of participants with DH were male (65%) with a median (IQR) age of 44 (33–60) years (Table 1). Among the participants who had urine *Histoplasma* antigen testing performed, 78/79 (99%) were positive. Most, but not all, people had an underlying immunosuppressed condition. Sixty-five (59%) were people living with HIV/AIDS; 14 (13%) people had solid organ transplants; 11 (10%) were people living with autoimmune disease, and 6 (5%) were people with active malignancy. The remaining 14 (13%) did not have any known underlying predisposition. Sixty-four out of 65 people living with HIV had a CD4 count < 200 cells/mm^3^, with a median (IQR) of 20 (10–38) cells/mm^3^. Seven people with autoimmune disease were on a tumor necrosis factor alpha inhibiting biologic agent (adalimumab or infliximab).

**Table 1.** Demographics and Underlying Conditions of Participants With Disseminated Histoplasmosis.

Demographics	Total (*n* = 110)
Median age, years, (Q1–Q3)	44 (33–60)
Male sex, *n* (%)	72 (65)
Caucasian race, *n* (%)	56 (51)
HIV/AIDS, *n* (%)	65 (59)
Median CD4 count, cells/mm^3^, (Q1–Q3)	20 (10–38)
Solid organ transplant, *n* (%)	14 (13)
Kidney	10 (9)
Kidney and pancreas	1 (1)
Kidney and heart	1 (1)
Heart	1 (1)
Liver	1 (1)
Autoimmune disease, *n* (%)	11 (10)
Rheumatoid arthritis	4 (4)
Lupus	3 (3)
Psoriasis	2 (2)
Ankylosing spondylitis	1 (1)
Crohn's disease	1 (1)
Malignancy^a^, *n* (%)	6 (5)
None identified, *n* (%)	14 (13)

^a^Malignancy includes 2 participants with diffuse large B-cell lymphoma, 2 with multiple myeloma, 1 with cutaneous T-cell lymphoma, and 1 with neuroendocrine tumor.

Twenty-two (20%) participants with DH met criteria for HLH based on HLH-2004 criteria (Table 2). The number of criteria assessed for each participant was variable. Temperatures, splenomegaly, and pancytopenia were assessed in nearly every patient, whereas natural killer cell activity and soluble interleukin-2 receptor levels were assessed in very few participants. Among the 22 participants with HLH, 6, 5, 8, and 3 participants had results for 5, 6, 7, and 8 of the HLH criteria, respectively. Hemophagocytosis was noted on 6/11 (55%) tissue biopsies for people with HLH. There was no statistically significant difference in age, sex, race, HIV/AIDS status, hospital length of stay, or in-hospital mortality/transition to hospice between those with HLH and those without HLH (Table 3). Participants with HLH were significantly more likely to be treated in the ICU. Seventeen people with DH did not require hospital admission. Participants with HLH were significantly more likely to have serum ferritin above the limit of quantification (LOQ), (>15 000 ng/mL), urine *Histoplasma* antigen above the LOQ, (>19 ng/mL), and serum BDG above the LOQ, (>500 pg/mL). The in-hospital mortality or discharge to hospice for people meeting HLH criteria was 18% (4/22, including 1 hospice discharge) and 7% (5/88, including 2 hospice discharges) in the non-HLH group. This difference was not statistically significant.

**Table 2.** Results of HLH-2004 Criteria Assessment in Participants With HLH, Those With Less Than 5/8 HLH Criteria (Non-HLH), and the Total Group of Participants With DH.

…	HLH (*n* = 22)		Non-HLH (*n* = 88)		Total (*n* = 110)	
HLH-04 Criteria	No. Positive/No. Tested	% Positive	No. Positive/No. Tested	% Positive	No. Positive/No. Tested	% Positive
Fever > 38.5 C	21/22	95.5	54/88	61.4	75/110	68.2
Splenomegaly^a^	14/22	63.6	19/88	21.6	33/110	30.0
Bicytopenia	22/22	100.0	58/86	67.4	80/108	74.1
Ferritin >500 ng/mL	22/22	100.0	38/48	79.2	60/70	85.7
Hypertriglyceridemia and/or Hypofibrinogenemia	21/22	95.4	16/51	31.4	37/73	50.7
Hemophagocytosis	6/11	54.5	1/23	4.3	7/34	20.6
Low NK activity	5/8	62.5	0/1	0	5/9	55.6
Elevated sIL-2 rec/CD25	12/12	100.0	0/0	N/A	12/12	100.0

^a^Splenomegaly was assumed negative if not explicitly positive on imaging or physical exam.

**Table 3.** Demographics, Clinical Features, and Laboratory Features of Participants With Disseminated Histoplasmosis.

Demographics, Clinical Features	HLH (*n* = 22)	Non-HLH (*n* = 88)	OR (95% CI)	*P*-value^a^
Median age, years, (Q1–Q3)	39 (33–53)	46.5 (34–61)		.2259
Male sex, *n* (%)	16 (73)	56 (64)	1.52 (0.54–4.29)	.4246
Caucasian race, *n* (%)	10 (45)	46 (52)	1.31 (0.51–3.36)	.5678
HIV/AIDS, *n* (%)	15 (68)	50 (57)	1.63 (0.60–4.39)	.3349
Admitted to hospital, *n* (%)	22 (100)	71 (81)	11.0 (0.64–190.56)	.0990
Median length of stay, days (Q1–Q3)	11 (8–24)	12 (7–23)		.6634
Treated in ICU, *n* (%)	13 (59)	28 (32)	3.10 (1.18–8.09)	.0210
In-hospital death or discharge hospice, *n* (% admitted)	4 (18)	5 (7)	2.93 (0.71–12.07)	.1359
Laboratory Features				
Median serum ferritin, ng/mL (Q1–Q3)^b^	15 000 (5498–15 000)	1013 (551–3607)		<.001
Serum ferritin >15 000 ng/mL, *n* (% tested)	15 (68)	7 (15)	12.6 (3.77–41.80)	<.001
Median beta-D-glucan, pg/mL (Q1–Q3)^c^	500 (134–500)	177 (56–463)		.0145
Serum beta-D-glucan >500 pg/mL, *n* (% tested)	10 (59)	11 (20)	4.50 (1.41–14.34)	.0110
Median urine *Histoplasma* antigen, ng/mL (Q1–Q3)^d^	19 (19–19)	11.0 (2.9–19)		.0028
Urine *Histoplasma* antigen > 19 ng/mL, *n* (% tested)	15 (79)	20 (33)	7.5 (2.20–25.57)	.0013

Q1–Q3, interquartile range; OR, odds ratio.

^a^Fisher's exact testing and Mann–Whitney U test were used for *P* values.

^b^Serum ferritin levels above 15 000 ng/mL were censored at 15 000.

^c^Serum beta-D-glucan (Fungitell, Associates of Cape Cod) values above 500 pg/mL were censored at 500.

^d^Urine Histoplasma antigen values above 19 ng/mL were censored at 19.

All 22 people meeting criteria for HLH received liposomal amphotericin B as initial treatment for DH. Of those hospitalized participants who did not have HLH, 52 out of 71 (73%) also received liposomal amphotericin B as initial treatment. All 17 participants with DH who did not require hospitalization received azoles as initial treatment. Table 4 shows demographic information, HLH directed therapies, and outcomes for participants with HLH. All participants either had AIDS or were taking immunosuppressive medications at the time of diagnosis of HLH. Fourteen (93%) of the 15 participants with AIDS survived, and one was discharged to hospice care. Four (57%) of the 7 participants without AIDS survived. This difference in mortality is not statistically significant (OR 10.5, *P* = .0676). Fourteen (64%) of the 22 participants with HLH received corticosteroids during hospitalization. The indications, specific agents used, and the doses of corticosteroids varied greatly. Three participants continued to receive their home dose of prednisone 5–10 mg per day throughout hospitalization. Four participants with AIDS (6, 8, 13, and 17) were started on prednisone at the time of admission to the hospital when the diagnosis of *Pneumocystis jirovecii* pneumonia was being considered. Participant 3, who survived, also received a dose of intravenous immune globulin and 9 doses of etoposide. Participant 12 received 2 doses of etoposide, and participant 14 received a dose of intravenous immune globulin; both of these participants died.

**Table 4.** Demographic Information, Therapeutic Interventions, and Outcomes for Participants With Histoplasmosis-associated HLH.

*P*	Age, Years	Sex	Underlying Condition (IS Medications)	HLH Criteria (No. Positive/No. Tested)	Corticosteroids^a^	Other HLH-directed Therapy	Outcome
1	53	M	AIDS	5/7	HC 50 mg iv q6 h for 3 days, HC 25 mg iv q6 h for 2 days, HC 25 mg iv q12 h for 6 days	None	Survival
2	49	M	Kidney transplant (pred, TAC, MMF)	7/7	HC 50 mg iv q8 h for 1 day, HC 50 mg iv q6 h for 11 days, HC 50 mg iv q8 h for 3 days	IVIG 1000 mg/kg once	Survival
3	33	F	AIDS	7/8	HC 50 mg iv q6 h for 3 days, then a decrease in daily dose over 10 days to 10 mg iv q12 h then a 5-day break before beginning dex^b^	IVIG 1000 mg/kg once; Etoposide 120 mg/m^2^ every 3 days for 4 doses, followed by every 7 days for 5 doses	Survival
4	40	M	AIDS	5/7	None	None	Survival
5	38	F	AIDS	5/6	None	None	Survival
6	35	M	AIDS	5/5	Pred 40 mg BID for 5 days	None	Survival
7	35	F	Kidney/pancreas transplant (pred, TAC, SRL)	5/5	Continuation of home prednisone 5 mg QD	None	Survival
8	33	M	AIDS	5/6	Pred 40 mg BID for 3 days	None	Survival
9	22	F	AIDS	5/6	None	None	Hospice
10	34	M	AIDS	6/6	None	None	Survival
11	35	M	AIDS	6/7	None	None	Survival
12	74	F	RA (pred, abatacept)	6/7	Pred 20 mg QD for 4 days, dex 20 mg iv QD for 5 days	Etoposide 150 mg/m^2^ for 2 doses	Death
13	21	M	AIDS	5/5	Pred 40 mg BID for 6 days, HC 100 mg iv Q8 2 days	None	Survival
14	41	F	DLBCL (R-CHOP)	5/7	HC 100 mg iv q8 h for 4 days, followed by dex^c^	IVIG 300 mg/kg	Death
15	65	M	RA (pred, infliximab, azathioprine, methotrexate)	5/6	Continuation of home pred 5 mg QD, dex 4 mg iv once	None	Survival
16	21	M	AIDS	8/8	None	None	Survival
17	30	F	AIDS	7/8	Pred 40 mg BID for 13 days, pred 20 mg BID for 4 days, pred 20 mg QD for 4 weeks	None	Survival
18	41	M	AIDS	6/7	None	None	Survival
19	51	M	AIDS	5/5	None	None	Survival
20	25	M	AIDS	5/5	HC 100 mg iv q8 h for 1 day	None	Survival
21	62	F	Kidney transplant (pred, TAC)	5/5	Continuation of home prednisone 5 QD	None	Survival
22	60	M	Kidney transplant (pred, TAC, EVL)	5/7	Continuation of home prednisone 5 QD, followed by HC 50 mg iv q6 h for 7 days until death	None	Death

Abbreviations: *P*, participant number; IS, immunosuppression; HC, hydrocortisone; IVIG, intravenous immune globulin; dex, dexamethasone; pred, prednisone; TAC, tacrolimus; MMF, mycophenolate mofetil; SRL, sirolimus; RA, rheumatoid arthritis; DLBCL, diffuse large B-cell lymphoma; R-CHOP, cyclophosphamide, doxorubicin, vincristine, and prednisone; EVL, everolimus.

^a^Dosing is oral unless specified as iv (intravenous). For each participant drugs and dosages given are listed in chronologic order.

^b^Dex 15 mg (10 mg/m^2^) for 7 days, dex 10 mg (6.75 mg/m^2)^ for 14 days, dex 8 mg (5.4 mg/m^2^) for 14 days, dex 4 mg (2.7 mg/m^2)^ for 77 days.

^c^Dex 4 mg iv q12 h for 1 day, dex 6 mg iv q12 h for 1 day, dex 6 mg iv qd for 3 days, dex 4 mg iv QD for 1 day.

## DISCUSSION

In this retrospective, single-institution study spanning 9 years, we identified 110 adult participants with DH, 22 of whom had HLH. We included people with HIV and those on immunosuppressants or biologic response modifiers in the setting of both solid organ transplantation and autoimmune disease. The HLH prevalence of 20% (24% for the subset of participants who were admitted to the hospital) is likely an underestimate because most participants were not assessed for every HLH-2004 criterion.

We diagnosed HLH among participants using the 8 clinical, biochemical, and histopathologic characteristics of HLH-2004 criteria, in accordance with consensus statements of the National Histiocyte Society [9]. Historically, the first HLH-1994 criteria was proposed and validated in the pediatric population with subsequent extrapolation to adults, with support of consensus statements [9, 17, 18]. These criteria were further refined in the HLH-2004 and, most recently, in the HLH-2024 criteria which updated the approach for familial HLH [19]. The eponymous finding of hemophagocytosis on cytology was subsequently found to be neither a sensitive nor specific finding in HLH and is not a requirement for diagnosis [20]. The HScore test was later developed as a diagnostic tool for identifying HLH in adults. External validation studies have shown similar sensitivity between HLH-2004 criteria and HScores, however the optimal cut-offs have been controversial [21]. A study of adults with HLH in an ICU setting concluded 4 criteria are sufficient as opposed to the original guidelines of 5 out of 8 criteria [22, 23]. A HScore cut-off of 169 has historically been used, but an external validation with a multicenter approach showed reduced specificity of 71% at this cut-off [21]. Currently, the Histiocyte Society recommends the use of HLH-2004 criteria (with clinical judgement and patient history) for the diagnosis of HLH in adults and recognizes the HScore as a supplemental diagnostic tool [9].

Our HLH prevalence of 20% is lower than the 36.1% found by Cruz-Quezada and colleagues in their study of HLH in people with HIV and DH in Mexico [5]. These authors used a broader definition of HLH: 5 of 8 HLH-2004 criteria, or a HScore of ≥169. Less than half of their participants with HLH met HLH-2004 criteria. Another methodologic difference is we did not restrict our study population to people with HIV.

We compared the demographics, clinical features, laboratory characteristics, and outcomes of those who met HLH-2004 criteria to those who did not. There were no statistically significant differences in demographic characteristics between the HLH and non-HLH group. A greater percentage of participants with HLH had HIV compared to those without HLH (68% vs 57%), but this difference was not statistically significant.

The extent of ferritin elevation in HLH is controversial. In the pediatric population, serum ferritin levels above 10 000 ng/mL have sensitivities and specificities over 90% for HLH [24]. The HLH-2004 criteria include serum ferritin levels above 500 ng/mL as consistent with HLH. However, hyperferritinemia alone has been shown to have poor specificity for HLH. In a 2015 review, only 19% of 113 adults with serum ferritin over 50 000 ng/mL had HLH [25]. A 2013 retrospective study at a single institution had 627 patients with ferritin levels greater than 1000 ng/mL, only 6 of whom had HLH, juvenile rheumatoid arthritis, or Still's disease [26]. In our study, a serum ferritin >15 000 ng/mL was strongly associated with the presence of HLH with an odds ratio of 12.6. We could not evaluate associations between HLH and higher ferritin levels because the upper limit of quantification is 15 000 ng/mL at our institution. Regardless, hyperferritinemia should prompt assessment of other HLH diagnostic criteria, especially in a person with underlying immune dysregulation.

We found statistically significant differences in both the serum BDG, and the urine *Histoplasma* antigen between people with HLH and without HLH. Sensitivity of BDG for histoplasmosis has been estimated at 87% with a specificity of 68% [27]. Urine *Histoplasma* antigen testing has a sensitivity of 93% in immunocompromising conditions for the diagnosis of DH [28]. Previous studies on HLH in DH have noted urinary *Histoplasma* antigens above the limit of quantification [29]. We found that urine *Histoplasma* antigen above the limit of quantification of 19 ng/mL had an odds ratio of 7.5 for HLH. To our knowledge, our study is the first to compare BDG and urinary *Histoplasma* antigens in people with and without HLH in DH. Our interpretation is that these biomarkers represent an increased organism burden, which increases the immunologic demand, subsequent cytokine activity, and risk for HLH in these patients. We recognize that BDG elevation alone is not specific and would be elevated in concomitant opportunistic infections such as candidiasis and pneumocystis, or from noninfectious sources such as cellulose dialyzers, intravenous immunoglobulins, human albumin, and surgical gauze [30].

All 22 participants with HLH received liposomal amphotericin B as induction treatment for DH. One participant received etoposide, two received IVIG, and one received both etoposide and IVIG for treatment of HLH. This is in part due to under-recognition of HLH at the time of hospitalization, and the care team's decision to treat with liposomal amphotericin B alone, which is consistent with the observation that most HLH induced by intracellular pathogens, like histoplasmosis, responds to specific antimicrobial therapy [1]. Given the diversity of therapeutic interventions that might have attenuated the course of HLH in our study population, we cannot draw any conclusions about the benefits or risks of corticosteroids, etoposide, or intravenous immune globulin. Furthermore, it is likely the more severely ill participants and those who were not improving received more aggressive therapy.

Participants with HLH were more likely than those without HLH to require intensive care, with a higher mortality outcome of 18% and 7%, respectively; a difference which did not reach statistical significance. With a larger sample size we may have shown a significant difference in mortality. ICU status is a potential confounding factor of mortality and HLH status. It is unclear whether the severity of underlying illness increases susceptibility of HLH and subsequent mortality, or if HLH alone contributes to severity of illness and mortality. Illness severity could bias the diagnosis of HLH as well with increased diagnostic testing or higher suspicion for HLH in critically ill patients. We suspect increased organism burden with higher urine *Histoplasma* antigens and BDG could also be contributing to mortality in the HLH cohort. Similarly, the 17 participants without HLH who did not require hospital admission would be expected to have lower mortality and lower risk of developing HLH. The mortality in our HLH group is low compared to prior studies [5, 29, 31]. However, we defined mortality as in-hospital mortality or discharging to hospice given the variability of follow-up time post discharge and the unpredictability of follow-up in our patient population. Deaths may have occurred after discharge from the hospital. All participants with HLH were treated with liposomal amphotericin B, which is superior to amphotericin B deoxycholate for the treatment of DH in people living with AIDS [32]. In contrast, the Cruz-Quezada study, wherein induction therapy for DH was either amphotericin B deoxycholate or itraconazole, had a 30-day mortality rate of 35% [5]. We show no statistically significant differences in HIV status between those with HLH and participants without HLH. Furthermore, among participants with HLH, the observed mortality was not statistically different in people with AIDS compared to people without AIDS; however, we recognize that our case numbers are too small and cofounders too numerous to make inferences on the prognosis and outcomes of histoplasmosis-associated HLH in people with AIDS compared to those without AIDS.

Our study has several limitations, including being a single institution, retrospective study. Diagnostic testing and therapeutic interventions were not standardized. The number of diagnostic tests performed for each participant was variable (Table 2). We categorized patients with less than 5 HLH criteria checked as non-HLH, as opposed to removing them from inclusion in the study. This means there are likely participants with HLH that were classified as non-HLH, which would underestimate the observed prevalence of HLH and overestimate the mortality in the HLH cohort in this study. Despite these limitations, we identified 22 participants with histoplasmosis associated HLH which is the largest cohort of adults with DH meeting the HLH-2004 criteria at a single institution thus far.

In summary, we show HLH is a common complication of DH. Serum ferritin >15 000 ng/mL, urine *Histoplasma* antigen above the LOQ, and serum BDG above the LOQ are markers for HLH and should prompt investigation for HLH. Most people with HLH due to DH that is treated with liposomal amphotericin B will survive to hospital discharge. Our results do not shed any light on whether or not anti-HLH therapies are beneficial. Further studies are needed to assess optimal treatment strategies in this population.
